# Editorial: Edible Insects: From Farm to Fork

**DOI:** 10.3389/fnut.2022.843302

**Published:** 2022-02-03

**Authors:** Mauro Serafini

**Affiliations:** Faculty of Biosciences and Technologies for Agriculture, Food and Environment, University of Teramo, Teramo, Italy

**Keywords:** edible insects, entomophagy, functional food, sustainability, consumer acceptance, novel foods, insect farming

Entomophagy, the practice of eating insects and invertebrates, has accompanied the human history through the centuries, playing a significant role in cultural and religious practices. Recently, a new global interest in edible insects and invertebrates arise from the impellent necessity of preserving agriculture resources to feed the 9 billion world's population predicted for 2050 and to obtain a drastic reduction of the ecological impact of food from animal sources. Under an ecological point of view, insects are characterized by a negligible ecological impact, in terms of carbon, water and ecological footprints. On the basis of their “ingredient” composition, edible insects are characterized by an excellent pattern of proteins, polyunsaturated fatty acids, vitamins and minerals. Moreover, a recent study provided evidences about the remarkable antioxidant content of antioxidant bioactive molecules, suggesting for edible insects a functional role in modulating oxidative stress, confirming antique use in traditional medicine from indigenous populations. Notwithstanding insects are part of the world's common diet of at least 2 billion people, in western countries characterized by an high food's ecological impact, entomophagy is often associated with entomophobia. Little is known about the food chain, leading edible insects from farm to plate, and on their role in human and planet wellbeing.

The aim of this Research Topic was to provide the reader with an extensive picture of the state of art about the ecological impact of edible insects, nutritional and functional properties, together with a better understanding of insects physiology and biochemistry, practices of insects farming and consumer's acceptance.

The work by Murugu et al. focus on the importance of edible insects like crickets, as alternative and valid source of classic nutrients such as protein, minerals and vitamins. Yu et al. shows the importance of understanding the changes of nutritional ingredients during the different developmental stages of *Tenebrio Molitor*. The review paper by Estrada et al. and D'Antonio et al. provide an overview of the benefits and challenge from food industry in the incorporation of insects in food products and review all the evidences about the antioxidant role of edible insects in different experimental models, respectively. As perspective The work by Doi et al. provide an interesting perspective on the area of the world that may be utilized for increased insect production under future climate changes.

In conclusion, we think that the Research Topic could foster the research of the scientific community on edible insects, amplifying the knowledges ad ensuring a correct information from the media to the consumers about the nutritional, functional and ecological importance of edible insects as potential ingredients for novel food development.

## Author Contributions

The author confirms being the sole contributor of this work and has approved it for publication.

## Conflict of Interest

The author declares that the research was conducted in the absence of any commercial or financial relationships that could be construed as a potential conflict of interest.

## Publisher's Note

All claims expressed in this article are solely those of the authors and do not necessarily represent those of their affiliated organizations, or those of the publisher, the editors and the reviewers. Any product that may be evaluated in this article, or claim that may be made by its manufacturer, is not guaranteed or endorsed by the publisher.

